# Nutraceuticals as Modulators of Autophagy: Relevance in Parkinson’s Disease

**DOI:** 10.3390/ijms23073625

**Published:** 2022-03-26

**Authors:** Michał Rakowski, Szymon Porębski, Agnieszka Grzelak

**Affiliations:** 1 The Bio-Med-Chem Doctoral School of the University of Lodz and Lodz Institutes of the Polish Academy of Sciences, University of Lodz, 90-237 Lodz, Poland; 2 Cytometry Lab, Department of Molecular Biophysics, Faculty of Biology and Environmental Protection, University of Lodz, 90-236 Lodz, Poland; szymon.porebski@edu.uni.lodz.pl (S.P.); agnieszka.grzelak@biol.uni.lodz.pl (A.G.)

**Keywords:** nutraceuticals, vitamins, autophagy, neurodegeneration, Parkinson’s disease

## Abstract

Dietary supplements and nutraceuticals have entered the mainstream. Especially in the media, they are strongly advertised as safe and even recommended for certain diseases. Although they may support conventional therapy, sometimes these substances can have unexpected side effects. This review is particularly focused on the modulation of autophagy by selected vitamins and nutraceuticals, and their relevance in the treatment of neurodegenerative diseases, especially Parkinson’s disease (PD). Autophagy is crucial in PD; thus, the induction of autophagy may alleviate the course of the disease by reducing the so-called Lewy bodies. Hence, we believe that those substances could be used in prevention and support of conventional therapy of neurodegenerative diseases. This review will shed some light on their ability to modulate the autophagy.

## 1. Introduction

People are undoubtedly living more comfortably than a century ago. Improved healthcare has led to a longer life expectancy, but a longer life has led to a higher rate of neurodegenerative diseases (NDs). The two most common NDs are Alzheimer’s disease (AD) and Parkinson’s disease (PD). It is thought that in the next 25 years, the number of people suffering from these diseases will double [1], and thus, the prevention of those diseases is indeed crucial. PD is caused by protein aggregates that form in the substantia nigra (SN) of the brain. These aggregates are often called Lewy bodies (LBs), and mainly consist of the α-synuclein (α-syn) and phosphorylated tau proteins (p-tau). Protein deposits disrupt the functions of the cell, eventually leading to its death. The neurons of PD patients contain protein aggregates, clustered organelles, and the remains of lysosomes and autophagosomes inside them [2], suggesting that the mechanisms responsible for the degradation of these redundant cell structures are defective. This defectiveness may be caused by protein aggregates. A high percentage of clustered organelles are mitochondria [2], once again suggesting that oxidative stress is mainly responsible for the neurodegenerative changes in neurons. In fact, PD’s etiology is very complex and primarily involves oxidative stress, the formation of protein aggregates (accelerated by oxidative stress), the disruption of calcium metabolism, mitochondria damage, gene mutations (especially mutations of the *SNCA* gene), and ineffective autophagy [3]. All these changes lead to the disruption of dopamine metabolism, causing dopamine deprivation, which results in the development o PD or other NDs.

### 1.1. Autophagy

The autophagy process is recruited for the degradation of certain parts of the cell; however, the molecular machinery behind it is much more complex and requires specific mediators. Along with proteasomes, it represents one of two major degradative pathways in eukaryotic cells. Several types of autophagy have been described, and they all have one thing in common: they transport the desired material to the lysosome. The type of transported material and the way in which this transport takes place define the type of autophagy.

Macroautophagy (MA) is the most commonly studied type of autophagy. In fact, due to its prevalence, it is often referred to as “the autophagy”. Briefly, at the beginning of MA, an autophagosome, a membrane-enclosed cellular organelle, is formed around the material that has been tagged for degradation. Then, the autophagosome transports the desired material to the lysosome and an autolysosome is formed as a result of direct fusion of the membranes of the two organelles. The autolysosome then degrades both the material supplied by the autophagosome and the autophagosome itself. Mechanistically, the biological processes involved in macroautophagy are very intricate. mTOR (mammalian target of rapamycin) complex 1 (mTORC1) is a central subunit of this pathway. mTORC1 phosphorylates the ULK1 (Unc51-like kinase) complex, thereby preventing it from initiating autophagosomal membrane formation and elongation by recruiting a ubiquitinating system containing LC3 (microtubule-associated protein 1A/1B-light chain 3) and Beclin-1 [4]. ULK1 is a serine/threonine protein kinase that is activated via phosphorylation and regulates the formation of autophagophores, which are the precursors of autophagosomes. Interestingly, ULK1 plays an important role in neuronal differentiation and is required for granule cell axon formation [5]. Proteins from the LC3 family are involved in elongation of the autophagophore membrane. Additionally, LC3 is involved in mitophagy, a specific process that removes the unwanted or damaged mitochondria [6]. Then, Beclin-1 acts as a core subunit of the PI3K (phosphoinositide 3-kinase) complex that exists in 2 main forms: PI3KC3-C1 (phosphatidylinositol 3-kinase catalytic subunit type 3) that is involved in the initiation of autophagosomes, and PI3KC3-C2, involved in maturation of the autophagosome and endocytosis [7,8,9]. In neurons, the end of the axon is the preferred site of autophagosome formation, from where it is transported into the cell’s lumen [10].

The conversion rate of LC3 protein has become the gold standard when measuring the activity of the autophagy process. Intracellularly, this protein is found in two forms: cytosolic (LC3-I) and lipid form (LC3-II). The lipid form is conjugated to phosphatidylethanolamine during the formation of autophagosomal membranes [11]. The activation of autophagy is thus positively correlated with the conversion of LC3-I to LC3-II, and the LC3-II/I rate is widely used as an autophagy marker. LC3-II is then degraded after fusion of the autophagosome with the lysosome; however, it has been proposed that LC3-II can be delipidated by Atg4 and recycled for subsequent autophagosome biogenesis [12].

Although the macroautophagy process seems quite simple, mechanistically, it is a very complex process. For example, even LC3 recruitment depends on at least seven proteins (Atg3, Atg4B, Atg5, Atg7, Atg10, Atg12, and Atg16) [13]. Hence, a master regulator of autophagy is needed to command all of those processes under different conditions, whether in health or disease. Transcription factor EB (TFEB) is one of the master regulators of autophagy, and it has been suggested as a possible new therapeutic target of PD [13,14,15]. TFEB has been shown to bind to a promoter motif that is responsible for activating the expression of autophagy and lysosomal genes [16]. Additionally, it stimulates autophagosome formation and its further fusion with the lysosome [17]. Furthermore, TFEB has the ability to enhance cellular clearance through lysosomal exocytosis mediated by the Ca^2+^ channel, suggesting its possible role in the development of PD [18].

Besides MA, both chaperone-mediated autophagy (CMA) and microautophagy can be additionally distinguished. CMA is a selective process and unlike MA, no transport vesicle is formed during it. In this case, the cargo is transported by a chaperone protein-heat shock protein 70 (Hsc70). Hsc70 recognizes the KFERQ sequence and once recognized, the substrates are translocated one by one to the lysosomal membrane, where they interact with Lamp2a (lysosome membrane-associated protein type 2) [19]. The efficiency of CMA depends on the amount of Lamp2a, the folding/decomposition rate of the translocational complex regulated by Gfap (glial fibrillary acidic protein) and EF1α (elongation factor 1 alpha) [20], and the presence of lys-Hsc70 in the lumen of the lysosome [21]. The main drawback of CMA is that it cannot degrade protein oligomers due to their size. Thus, CMA could be important in the early stages of ND development, when the proteins have not yet aggregated into big clumps.

Microautophagy is a non-selective lysosomal degradative process, which is complementary to macroautophagy and CMA. However, since microautophagy targets rather small molecules, it is not thought to be important in neurodegenerative diseases caused by the aggregation of proteins.

### 1.2. Relevance of Autophagy Modulation in PD

As a result of advancing age and excess oxidative stress, the efficacy of autophagy and proteasomes decreases, leading to the accumulation of oxidized proteins. The first reports on α-syn degradation proposed a mechanism based on the action of the ubiquitin-proteasome axis as being responsible for this process [22], although newer data suggests that the lysosomal pathway may be the main process responsible for α-syn degradation in neurons [23]. Indeed, in vivo lysosomal degradation is much more important when α-syn is overexpressed [24].

Interestingly, PD patients have an increased number of autophagosomes in the brain tissue compared to healthy subjects [25]. An increased accumulation of lipid forms of LC3 (LC3-II) has been confirmed during PD, indicating autophagosome formation [26]. Decreased levels of Atg7 (autophagy related 7) and increased levels of mTOR have been demonstrated in patients with Lewy body disease and in a model of α-synucleinopathy [27], suggesting possible impairment of autophagy. Additionally, it has been reported that α-syn can interfere with the early stages of autophagosome formation via inhibition of Rab-1a (Ras-related protein 1A), leading to mislocalization of Atg9 through deregulation of HMGB1 (high-mobility group box 1) or by the interaction of JNK1 (c-jun N-terminal kinase 1) with Beclin-1 [28]. Thus, the accumulation of α-syn is cytotoxic for the neurons and can also cause a disruption of the autophagy process, leading to even faster accumulation of proteins.

The CMA has been suggested as the major pathway responsible for removal of wild type α-syn in neurons [29]. However, the CMA degrades only monomers or dimers of α-syn. Oxidation or nitration of α-syn reduces the efficiency of CMA and phosphorylation or dopamine modification of α-syn completely blocks its elimination by CMA [30]. Thus, the disruption of the CMA function may be the reason for α-syn accumulation in Parkinson’s disease [31], although most likely only during the developmental stage of the disease. Early diagnosis seems to be crucial, as clinical induction of autophagy would probably be most effective in the early stages of PD, when protein deposits are small enough to be removed by autophagy and extensive nerve cell damage has not yet occurred. 

Dopaminergic neurons are metabolically active and require large amounts of energy from mitochondria, so they are highly susceptible to mitochondrial damage and inefficient removal of damaged organelles [32]. The accumulation of damaged organelles can lead to increased levels of reactive oxygen species, which can damage adjacent healthy mitochondria and greatly accelerate neurodegeneration. Ultrastructural characterization of autophagosomes in the brain of PD patients revealed many phosphor-ERK-labeled mitochondria, suggesting the presence of PD-related mitophagy [25]. Despite the physiological localization of α-syn, cellular stress and disease can cause translocation of this protein to the inner mitochondrial membrane, where it interacts with the first respiratory chain complex. This interaction results in decreased activity of the first complex and increased free radical generation in vivo [33]. Further investigation of dopaminergic neurons from mice overexpressing α-syn revealed the presence of α-syn monomers and oligomers localized on the mitochondrial membrane, which was correlated with an increased mitophagy rate [33]. α-syn oligomers have been shown to bind with high affinity to the mitochondrial TOM20 protein in post-mortem-collected tissue of PD patients and in animal models of PD [34]. The strong binding of TOM20 to α-syn prevents its interaction with the TOM22 receptor, resulting in defective protein import into mitochondria, which leads to a loss of mitochondrial membrane potential. The interaction of α-syn with TOM20 may later contribute to mitophagy activation [34]

In addition to the aforementioned TFEB, Nrf2 (nuclear factor erythroid 2-related factor 2) has been proposed as a possible therapeutic target in the treatment of PD. Nrf2 is a leucine-zipper transcription factor that binds the antioxidant response element (ARE) and thus regulates the expression of more than 100 genes, mainly encoding detoxifying enzymes and antioxidants [35]. Niu et al. [35] provided an extensive review on the modulation of Nrf2 in PD. They highlighted several natural products targeting Nrf2, for example, flavanones, such as naringin, hesperetin, ampelopsin, and pinocembrin, that act through the Nrf2/ARE signaling pathway in PD models [36], or carnosic acid [37] and berberine [38], both providing protection against 6-OHDA-induced cell death in PD models. Additionally, Niu et al. [35], in their review, presented some of the interactions involving Nrf2 in different PD models, ranging from MPTP-treated mice through to paraquat-treated *Drosophila melanogaster* to 6-OHDA-treated SH-SY5Y cells. Although Nrf2 in PD seems to mostly interact in an ARE-dependent manner (especially in MPTP-treated animals), there are also reports about its interactions with other mediators, such as NF-κB [39,40].

NF-κB (nuclear factor kappa B) is, in addition to TFEB and Nrf2, yet another transcription factor that has been proposed as a potential therapeutic target of PD. It is a ubiquitously expressed transcription factor that is responsible for maintaining cell survival, proliferation, activation of some of the anti-apoptotic pathways, and cellular response to inflammation [41]. Due to its multifunctionality, the involvement of NF-κB in pathways that relate to NDs is somewhat impressive: it has been reported to interact via TLRs (toll-like receptors), GSK-3β (glycogen synthase kinase 3β) and PI3K/Akt pathway, p38-MAPK pathway, and many more [41]. Furthermore, the absence of Nrf2 leads to the induction of NF-κB-dependent processes that promote neurodegeneration [42], albeit the presence of Nrf2 protects against MPTP-induced neurodegeneration via suppression of the NF-κB and p38/MAPK signaling pathways [43,44]. 

## 2. Modulation of Autophagy by Nutraceuticals: Support for the Treatment of NDs

Nutraceuticals are substances that combine nutritional value with the characteristics of pharmaceuticals. Nutraceuticals include all compounds of natural origin that have a positive effect on the human body accompanied by a nutritional value. According to the European Food Safety Agency, dietary supplements are concentrated sources of nutrients or other substances with a nutritional or physiological effect.

In this section, the ability of selected vitamins and nutraceuticals to modulate autophagy is analyzed. Additionally, their potential relevance to neurodegenerative diseases—especially Parkinson’s disease—is summarized. Despite the fact that some of those substances have no impact on PD alone (or have not been tested yet), they can probably be used as a supportive therapy with conventional drugs.

**Figure 1:**
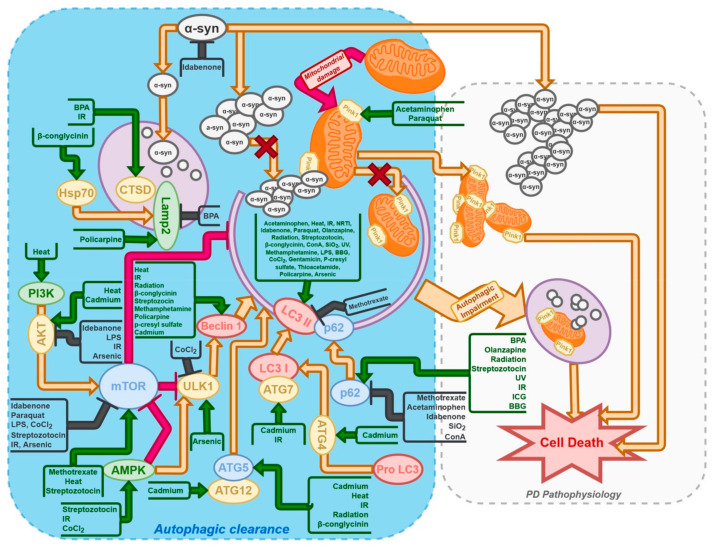
Diagram presenting modulation of the autophagy pathway in mammalian nerve cells induced by factors used to establish a research PD model. The green arrows indicate induction of mRNA/protein expression, whereas the red arrows indicate an inhibitory effect of the presented compounds. Additionally, the protein–protein interactions are shown, in which the yellow arrows indicate induction of mRNA/protein expression, whereas the black arrows indicate an inhibitory effect.

The response to nutraceuticals may vary between research models. This is because these models are usually established through chemical modulation of certain cellular processes. Figure 1 presents the influence of those models on the autophagy process in nerve cells. These interactions are crucial when assessing the role of certain nutraceuticals on PD and every other disease.

We did our best to select papers on the modulation of autophagy by selected nutraceuticals with relevance to neurodegenerative diseases (mainly PD); however, such papers were not always readily available. Thus, to reliably present their ability to modulate autophagy, data based on other models have also been included. We searched PubMed as the main source of knowledge, with Google Scholar, Web of Science, and Scopus as additional sources of data. Search criteria consisted of “*compound name*” AND “autophagy” AND “Parkinson’s disease” OR “neurodegeneration” OR “neurodegenerative disease” OR “α-synuclein” OR “synucleinopathy” OR “- ”. The selected results of this research are presented and described below.

### 2.1. Carotenoids and Retinoids

β-Carotene, a precursor of retinol (vitamin A; vitA), is an antioxidant that is supplied to the human body through the diet and can be found in a range of vegetables and fruits (mostly in carrots, mangoes, spinach, and corn) [45]. A prospective cohort study linked high amounts of β-carotene in the diet to a reduced risk of developing PD [46]. It has been suggested that continuous intake of antioxidant supplements, such as β-carotene, may significantly contribute to the health status of PD patients by stabilizing oxidative processes [45]. However, a meta-analysis published in *The Lancet* in 2004 found no protective properties of β-carotene against PD [47]. The same results were obtained in a study published 16 years later, which found no association between β-carotene levels and PD risk [48]. Overall, carotenoids are quite often researched in the matter of autophagy-modulating activity (Table 1).

All-trans retinoic acid (ATRA), the active metabolite of vitA, is used in dermatology and in the treatment of acute promyelocytic leukemia, in which it induces granulocytic differentiation of the blast, leading to the death of differentiated leukemic cells. Additionally, it mediates the induction of autophagic flux in ATRA-sensitive SKBR3 breast cancer cells through upregulation of LC3-II and β-catenin protein expression via activation of pan-retinoic acid receptor α [49]. 

Crocin, a carotenoid found in *Gardenia jasminoides Ellis* that has been used for centuries in traditional Chinese medicine, has been shown to have antiproliferative, anti-inflammatory, and antioxidant abilities [62]. Additionally, crocin downregulated autophagy in an ischemic model of HT22 cells, suggesting that the anti-ischemic effect of crocin may be mTOR dependent [50]. 

Lycopene, a precursor of β-carotene, is a bright red carotenoid that is mostly found in tomatoes and other red fruits and vegetables. Surprisingly, lycopene is the most prevalent carotenoid in the human diet [63]. Choi and Kim [64] recently published a review on the relevance of lycopene in pancreatitis, in which they concluded that lycopene, due to its antioxidative properties, could attenuate the severity of pancreatitis by preventing excess ROS-induced autophagy. Their conclusions were based on several papers, in which, for example, lycopene was reported to attenuate cadmium-induced activation of autophagy in murine hippocampal cells [52], rescue endothelial progenitor cells of type 2 diabetes mellitus (T2DM) rats from excess autophagy [53], and alleviate gentamicin-mediated cell death, apoptosis, and autophagy [54]. The reports suggest that lycopene could be used as a potent antioxidant, reversing excess autophagy flux.

Mutations in the *TBK1* (TANK-binding kinase 1) gene have been linked to amyotrophic lateral sclerosis (ALS) [55]. TBK1 regulates the phosphorylation of SQSTM1 (sequestosome 1; p62), which is a critical step in macroautophagy. Catanese et al. [55] reported that TBK1-mutant motoneurons accumulate immature phagophores due to a failure in the elongation phase, and 4-hydroxy-(phenyl)retinamide has been identified as a potent modifier of autophagy, worsening the autophagy status of cells by upregulating p62, with a simultaneous reduction in the level of Atg10.

Xanthophylls are compounds that belong to the carotenoid group. These yellow pigments are widely distributed among plants. Astaxanthin belongs to this family and has been reported to modulate the autophagy process in a range of experimental models (Table 1). However, in the vast majority of reports collected in a review by Kim and Kim [65], astaxanthin caused deactivation of autophagy, mainly via the PI3K/Akt (phosphatidylinositol 3-kinase/protein kinase B) and p38 (mitogen-activated protein kinase p38) pathways. Interestingly, astaxanthin has been shown to counteract the neurotoxicity induced by amyloid β [66] and 6-OHDA (6-hydroxydopamine) [67], which suggests its potential relevance in the therapy of neurodegenerative diseases. Additionally, astaxanthin has been shown to abolish *H. pylori*-induced apoptosis by activating autophagy via an AMPK-dependent mechanism [56]. Fucoxanthin, a carotenoid derived from marine algae, has been reported to upregulate the Atg7, p62, and LC3-II protein levels accompanied by downregulation of the Atg4B protein level in a human nasopharyngeal carcinoma C666-1 cell line [60].

Apparently, the use of dietary carotenoids in the treatment of NDs is often a topic of review papers. Cho et al. [68], in their review, listed carotenoids that are potentially relevant in ND therapy. Based on their paper, it seems that crocin [50], lutein [57,58,59], lycopene [52,54,64], astaxanthin [56,65], and fucoxanthin [61] could modulate autophagy under specific conditions, although the precise molecular mechanism of the autophagy-modulating activity of carotenoids remains unclear.

### 2.2. Ascorbic Acid

Ascorbic acid (vitC) is undoubtedly the most often researched (nearly 70,000 records in PubMed) vitamin. It features powerful antioxidant activity, preventing lipid peroxidation and mitochondrial damage. The main sources of vitC are fruits and vegetables, especially citrus fruits and strawberries. Dietary supplements containing vitC are also very popular, notably during the flu season.

The antioxidant activity of vitC has been confirmed in at least several hundred original papers. In March 2021, a paper was published that linked low levels of vitC with a higher prevalence of PD (*p* < 0.001) [69]. A meta-analysis of the effect of vitC and vitE supplementation on PD risk found no correlation of supplementation with vitC on PD risk [70]. A Mendelian randomization study examining the effect of genetically elevated plasma vitC levels in relation to the age of onset of first PD symptoms found no correlation between plasma vitC levels and the probability of developing PD [71]. However, it has been shown that people with genetically elevated plasma vitC levels (the following genes have been associated with higher plasma vitC levels: *SLC23A3*, *CHPT1*, *BCAS3*, *SNRPF*, *RER1*, *MAF*, *GSTA5*, *RGS14*, *AKT1*, *FADS1*) have a significantly lower age at the onset of PD when compared to a control group [71,72].

Oxidative stress underlies all neurodegenerative processes. The maintenance of intracellular redox equilibrium positively influences some of the diagnostic markers of PD progression. However, there is no evidence for the impact of vitC on the clinical course of this disease. Despite the numerous papers that have been published about vitC, there is not much data about its potential to modulate the autophagy process either, especially in PD (Table 2).

Nevertheless, some of the papers reported the ability of ascorbic acid to inhibit autophagy, for example, through reducing de novo synthesis of Beclin-1 in the pilocarpine-induced seizure rat model [73]. Pretreatment with L-ascorbate protected primary rat cortical neuron-glia cells from methamphetamine-induced neurotoxicity by reducing the autophagy markers, attenuating the production of ROS and expression of cleaved caspase-3 [75]. On the contrary, the paper by Martin et al. [76] reported that physiological concentrations of vitC accelerated the degradation of intra- and extracellular proteins targeting the lysosomal lumen by the autophagic pathway in human astrocyte glial cells. However, in bone marrow stromal cells, vitC has been reported to inhibit ROS-induced autophagy [74].

The mechanism behind vitC modulation of autophagy seems to be clear due to its antioxidant abilities. The lack of research on this topic confirms this hypothesis, especially when compared to the total number of original papers featuring vitC. Thus, this suggests that the only possible way to modulate autophagy using vitC is through the attenuation of oxidative stress. However, it does not mean that vitC is ‘useless’ in this matter, because the side effects of many drugs are ROS-based. Hence, vitC supplementation could attenuate such side effects and improve adherence to therapy.

### 2.3. Calciferol

Calciferol (vitD) is produced endogenously by skin cells exposed to UV-B radiation. Exogenously, it is most effectively supplied by dietary supplements because only a few foods contain a sufficient dose of vitamin D_3_ (e.g., tuna, carp, salmon, fatty cheese, and some forest mushrooms). Vitamin D_3_ (vitD_3_) is the most essential form of vitD due to the fact that it is involved in many intracellular signaling and metabolic pathways, e.g., calcium metabolism and bone growth. 

Neurochemical analysis of the effects of vitD_3_ in a mouse model of PD showed that vitD_3_ supplementation protects against oxidative stress and the loss of dopaminergic neurons when compared to a control [77]. Jang et al. [78] presented results from an in vitro study of a cellular model of PD (SH-SY5Y cells with rotenone-mediated neurotoxicity) that confirmed a reduction in ROS levels after vitD_3_ supplementation. They also noted an increase in the levels of the autophagy markers: LC3, Beclin-1, and AMPK, after treatment with vitD_3_. Based on these results, the authors concluded that incubation with rotenone caused a decrease in Beclin-1 protein levels and an increase in mTOR, an effect that was eliminated via incubation with vitD_3_. Many papers have assessed the role of vitD in the modulation of autophagy, some of which are summarized in Table 3.

The results of some clinical studies suggest that vitD_3_ may have a positive effect on the clinical course of PD. In 2008, Evatt et al. [86] reported that 55% of patients with PD were vitamin D_3_ deficient compared to 36% of individuals in the control population. Two years later, results from a cohort study of the Finnish population was published, indicating that higher plasma levels of 25-hydroxy-vitD (which is a physiological indicator of the vitD_3_ level) were correlated with a lower likelihood of developing PD [87]. These results suggest that vitD_3_ may exert a neuroprotective effect on dopaminergic neurons.

The pleiotropic effect that vitD exerts in the human body affects autophagic flux as well. There are plenty of sites where vitD could possibly modulate the autophagy process. For example, vitD has been found to attenuate particle-induced pulmonary damage and promote tissue repair via TGFβ1 (transforming growth factor beta 1) signaling pathway inhibition and upregulation of MMP9 (matrix metallopeptidase 9) expression [79]. Additionally, it modulated autophagy through the Nrf2 (nuclear factor erythroid 2-related factor 2)-dependent pathway through degradation of p62, thus resulting in decreased Nrf2 ubiquitination [79].

VitD is often used in therapies for microbial-induced diseases. It is considered as one of the therapeutical agents for the treatment of tuberculosis, and in fact, before the discovery of streptomycin, the treatment of tuberculosis consisted of vitD-rich cod liver oil supplementation and exposure to sunlight. Supplementation with vitD increases the cytoplasmic level of Ca^2+^ by expressing calcium-regulating proteins [88], leading to CaMK-β (Ca^2+^/calmodulin-dependent protein kinase II beta)-mediated activation of autophagy [89]. In addition, calcitriol causes a dose-dependent decrease in *Porphyromonas gingivalis* viability and promotes autophagy in U937-derived macrophages [80]. Furthermore, vitD deficiency has been associated with respiratory tract infections. A study by Dai et al. [84] showed that vitD-deficient rats that were infected with *Aspergillus fumigatus* exhibited a higher death rate and more fungal growth and weight loss than uninfected control animals. VitD lowered the *A. fumigatus*-induced autophagy level in cells, suggesting that vitD deficiency can be associated with the formation of excessive autophagy-induced lysosomes, leading to a higher rate of death [84]. VitD deficiency has also been observed during hepatitis C virus infection in patients with hepatocellular carcinoma; these patients had significantly lower serum LC3 protein levels than healthy subjects [85]. 

VitD supplementation enhances the regeneration of UV-injured skin via inhibition of inflammatory cytokines associated with enhanced autophagy in myeloid anti-inflammatory M2 macrophages [83]. In addition, biopsies from UV-exposed human skin revealed a similar increase in macrophage autophagy after vitD treatment [83]. Moreover, vitD induces autophagy in a streptozotocin (STZ)-induced type 1 diabetes mellitus mouse model while decreasing the apoptosis rate [81]. 

Tavera-Mendoza et al. [82] reported that the vitD receptor (VDR) acts as a master transcriptional regulator of autophagy. They observed that vitD supplementation induces autophagy in the normal murine mammary gland via upregulation of the LC3 protein level accompanied with an increased autolysosome volume [82]. This report could provide an explanation for the autophagy-modulating activity of vitD. Interestingly, according to Table 3, it can be concluded that vitD deficiency could result in downregulation of autophagy; thus, the level of vitD should be tested in those with PD, as low levels of vitD could possibly downregulate the autophagy process. Indeed, serum levels of vitD in PD patients have been found to be approximately two times lower than in healthy subjects, suggesting that there may be a correlation between the vitD level and autophagy in PD [90].

### 2.4. Tocopherols and Tocotrienols

Tocopherols (vitE) are fat-soluble compounds that exhibit antioxidant activity, of which α-tocopherol (α-tocph) is the main form. It scavenges free radicals, such as hydroxyl radical and peroxides, and inhibits lipid peroxidation [45]. 

To date, a number of clinical trials and in vitro studies have been conducted using vitE. VitE administered in combination with vitC slows the development of PD in the early stage by 2.5 years compared to the placebo group [45]. Based on nutritional questionnaires, higher vitE intake has been associated with a lower risk of PD [91]. Unfortunately, a clinical study led by Scheider et al. [92] reported no effect of vitE on clinical symptoms of PD and did not slow the disease progression either. Their results were consistent with results of the largest clinical trial on the effect of vitE on PD, called the Deprenyl and Tocopherol Antioxidative Therapy of Parkinsonism (DATATOP) [93]. In this study, selegiline was shown to delay the onset of PD symptoms while α-tocph showed no significant effect on the course of the disease. However, a meta-analysis from 2021 showed a reduction in the risk of developing PD in people taking vitE [70].

The mechanism of action of vitE is usually attributed to its antioxidative abilities, yet there are also papers reporting the ability of vitE to modulate the autophagy process, which is especially important during development of synucleinopathies. Hence, some of the reports on the modulation of autophagy by vitE are summarized in Table 4 and described below. 

CRMP-2 (collapsin response mediator protein 2) maintains the axonal conditions of neurons. Fukui et al. [94] found that vitE deficiency leads to lower expression of CRMP-2 and higher expression of the autophagy marker LC3 protein in the cerebral cortex and hippocampus of mice. Additionally, they found that the number of cells in the CA1 region of the hippocampus was lower in vitE-deficient mice when compared to the control [94]. A follow-up study showed no differences in axonal degeneration between short-term vitE-deficient mice and the control group [106]. However, when they compared the short-term (3 months) to long-term (6 months) vitE deficiency, they found that the expression of LC3-II is significantly higher in short-term than in long-term mice [106]. This suggests that autophagy is rapidly activated after the cellular deposits of vitE are finished off and it is downregulated when the cells are slowly recovering from the vitE outage.

Status epilepticus and seizures cause substantial generation of ROS; hence, vitE has been proposed as a remedy for this upregulation of oxidative stress. α-tocph has been reported to partially inhibit autophagy in rats with pilocarpine-induced status epilepticus [95]. The same research group found that vitE reduces pilocarpine-induced CMA upregulation [96]. Furthermore, antioxidants, such as vitE, could possibly inhibit ROS-induced CMA in neurons during seizures or status epilepticus. Their follow-up study [96] shed new light on the role of vitE in the development of PD, as CMA has been proposed as a mechanism of α-syn degradation [23]. 

Growing evidence suggests that iron deposition could indeed play a crucial role in the development of PD [107]. Wan and colleagues [108] showed on primary dopaminergic neurons and SH-SY5Y cells that iron loading increased the α-syn and ROS levels but did not change the α-syn mRNA level. Further experiments indicated that iron loading downregulated Beclin-1 levels and decreased the LC3-II/I ratio. VitE did not affect the iron-induced α-syn levels but significantly decreased ROS production [108]. The authors suggested that autophagy dysfunction may be the main reason for iron-induced α-syn accumulation in neuronal cells. VitE has been reported to alleviate the upregulation of autophagy in renal tubular epithelial cells of diabetic nephropathy patients and rats, and in HK-2 cells with advanced glycation end product-induced diabetes [97]. Moreover, it has been shown that diabetic nephropathy leads to the blockage of autophagosome lysosomal degradation, thus causing autophagosomal stress, which could be attenuated by vitE supplementation [97]. In addition to NDs, autophagy processes are also involved in mental diseases. For example, Huang et al. [98] studied the neuroprotective effect of α-tocph in chronic unpredictable mild stress (CUMS) mice, often used as an animal model of depression. Based on behavioral tests, they concluded that supplementation with α-tocph could ameliorate the deficits induced by CUMS. A follow-up experiment showed an increase in mTOR activity, which was alleviated by supplementation with α-tocph [98]. These results suggest that autophagy downregulation may be involved in the development of depression, thus restoration of autophagy flux could become a relevant therapeutic target.

Tocotrienols are vitE isomers that are rapidly gaining popularity as dietary supplements. Depending on the position and number of methyl groups on the chromanol ring, four isomers of tocotrienols can be distinguished: α, β, γ, and δ [100]. Lekli et al. [100] administered γ-tocotrienol (γ-toctr) with or without resveratrol to rats for 30 days. Then, isolated perfused hearts were subjected to ischemia followed by reperfusion. They found that γ-toctr upregulated the Beclin-1 and LC3-II/I conversion ratio; however, the synergistic effect of γ-toctr and resveratrol was approximately 2.5 times higher than γ-toctr or resveratrol alone [100]. This evidence clearly indicates that nutraceuticals should be studied in combination with several compounds, because their synergistic effect can be of benefit to patients. Karim and colleagues [99] obtained similar results on hepatocytes (H4-II-E cells) in which α-tocph significantly increased the cytosolic LC3-II/I ratio, suggesting that early autophagosome formation may be the main place where α-tocph exerts its action. Furthermore, γ-toctr has been reported to strongly increase the level of LC3-II in human prostate cancer cells [101]. Additional information was provided by Jang et al. [102], who showed that γ-toctr induced the expression of LC3-II in HCT-116 cells. Rickmann et al. [104] proposed a mechanism of action for tocotrienols, based on their research conducted on pancreatic stellate cells (PSCs). They reported that tocotrienol-rich fraction (TRF) from palm oil reduced the viability of activated PSCs via the induction of apoptosis, which was confirmed by an analysis of the LC3-II protein levels. Considering that TRF caused an intense and sustained mitochondrial membrane depolarization and cytochrome C release, and that cyclosporin A (used to block the mitochondrial permeability transition pore) completely attenuated the cytotoxic effect of TRF, the authors concluded that TRF exerts its cytotoxic action by targeting the mitochondrial permeability transition pore. Interestingly, γ-toctr has been reported to induce autophagy in mouse + SA, MCF-7, and MDA-MD-231 cell lines [103]. However, the same treatment with γ-toctr did not cause any significant changes in immortalized murine and human normal mammary epithelial cell lines (CL-S1 and MCF-10A) [103]. 

Naturally occurring vitE derivatives are not the only derivatives able to induce autophagy. Alpha-tocopheryloxyacetic acid (α-TEA) is a semi-synthetic vitE derivative that triggers autophagy in murine mammary and lung cancer cells, which was confirmed via an increase in autophagosome formation [105].

The ability of tocotrienols to induce autophagy is promising, especially their selective action on cancers rather than normal cells [103]. It suggests that vitE—especially tocotrienols—could activate autophagy, leading to the clearance of protein aggregates.

### 2.5. Coenzyme Q10

Coenzyme Q10 (CoQ10) exhibits antioxidant and cytoprotective effects. It is a component of the oxidative phosphorylation chain in mitochondria. The neuroprotective effect of CoQ10 has been confirmed in vitro. CoQ10 supplementation has been shown to inhibit the loss of dopamine and dopaminergic neurons in vivo in a mouse model of MPTP-induced PD [45]. In 2002, the results of a phase II clinical trial (prospective, randomized, double-blind, 40 participants) were published, showing that high doses of CoQ10 (1200 mg/day) are safe and well-tolerated by patients and resulted in an improvement of overall clinical symptoms [109]. Then, 1 year later, a clinical trial (single-center, simultaneous, placebo-controlled, double-blind) was conducted, during which participants were given 360 mg of CoQ10 daily for 4 weeks [110]. Results showed a moderate improvement in PD symptoms. In contrast to these reports, a paper from 2007 reported no benefit of a 3-month (1200 mg/day) CoQ10 supplementation in patients with PD [111]. A meta-analysis of the clinical trials conducted to date clearly established that CoQ10 is safe and well-tolerated by patients, although clear evidence for its therapeutic effect is lacking [112].

Some of the available data on the modulation of autophagy with CoQ10 are presented in Table 5. Briefly, in a paper from 2019, Mohamed and colleagues [113] showed that CoQ10 can attenuate methotrexate-induced cell loss from the liver and lungs by inducing autophagy. Furthermore, CoQ10, via a decrease in p62 and lysosomal protease-related genes (CTSB and CTSD) mRNA expression levels, restored the autophagy flux and lysosomal degradation pathways in bisphenol A-treated myoblasts (C2C12 cell line) [114]. Based on the cultured fibroblasts derived from a patient with MERRF syndrome (myoclonic epilepsy associated with ragged-red fibers), Villanueva-Paz et al. [115] showed that supplementation with CoQ10 alleviates MERRF-induced upregulation of the LC3-II level. The ratio of phospho-AMPK/total-AMPK was significantly increased in MERRF fibroblasts (250%), as presented by Villanueva-Paz et al., although phosphorylation of AMPK was further stimulated by CoQ10 [115]. Additionally, treatment with CoQ10 attenuated Parkin-mediated mitophagy by preventing its translocation to mitochondria. However, Zhang et al. [116] experimentally confirmed the ability of CoQ10 to induce mitophagy via the PINK1 (PTEN-induced kinase 1)-Parkin pathway in the stressed cells of a mouse with acetaminophen-induced liver injury. In addition, CoQ10 has been found to protect chicken myocardial cells during heat stress through deactivation of the PI3K/Akt/mTOR pathway and upregulation of autophagy-associated genes [117]. Supplementation of rats with acute myocardial ischemia-reperfusion injury with CoQ10 has been found to induce the autophagy [118].

Supplementation with CoQ10 indeed modulates the autophagy process; however, it has been also shown that CoQ10 deficiency could also lead to the activation of autophagy via overexpression of autophagy-associated proteins [124]. Additionally, augmented lysosomal activity and mitochondria-containing early autophagosomes have been found in fibroblasts from patients with a CoQ10 deficiency, clearly indicating the occurrence of mitophagy [124]. All the mentioned effects were alleviated with CoQ10 supplementation. MELAS (mitochondrial encephalomyopathy, lactic acidosis, and stroke-like episodes) is a mitochondrial disease mostly caused by point mutations in tRNA genes encoded by mtDNA. One of the markers of this disease is a lower basal level of CoQ10 in fibroblasts, leading to an impairment of autophagosome elimination [119]. It causes an accumulation of defective mitochondria in cells, suggesting an activation of mitophagy with deficient autophagic flux, which could be restored with CoQ10 supplementation [119].

Nucleoside reverse transcriptase inhibitors (NRTIs) are known for their antiretroviral activity, and thus have been used in the treatment of those infected with HIV-1. However, NRTIs most likely cause mitochondria injury in the endothelial cells of the host, possibly due to ROS generation [120]. Xue and colleagues [120] reported that supplementation with CoQ10 abolishes NRTI-induced mitochondria injury. Additionally, they found that CoQ10 prevents NRTI-induced overexpression of LC3, therefore protecting the mitochondria from degradation via mitophagy.

MitoQ, a CoQ10 derivative mainly targeting mitochondria, increased the expression of p-Akt, p-GSK-3β, and mTOR but decreased Beclin-1 and LC3-II/LC3-I in a rat model of sepsis-induced acute lung injury, leading to downregulation of autophagy [123]. Idabenone, a CoQ10 analogue often used in neurodegenerative research, promotes α-synuclein degradation via autophagy by suppressing the AKT/mTOR pathway in α-synuclein-overexpressing SH-SY5Y cells [125]. Furthermore, CoQ10 alleviates pancreatic fibrosis via the PI3K/AKT/mTOR signaling pathway, resulting in the downregulation of autophagy in primary pancreatic stellate cells isolated from C57BL/6 mice [121,122]. Interestingly, a natural CoQ10 derivative—antroquinonol—inhibits Akt phosphorylation at Ser^473^, which is critical for Akt activity, and mTOR phosphorylation at Ser^2448^, a site dependent on mTOR activity [126]. The authors [126] suggested that antroquinonol via PI3K/Akt/mTOR inhibition induces anticancer activity in human pancreatic cancer PANC-1 and AsPC-1 cells. 

CoQ seems to ideally support during ND therapy. Regrettably, the available data from in vitro research does not correlate with comprehensive meta-analysis papers of clinical trials. This is why more data is needed to reliably assess the relevance of CoQ supplementation in NDs. Although it is also crucial to test patients for a potential deficiency of a specific vitamin or nutraceutical because—especially in elders—this may lead to deterioration of their health status.

### 2.6. Curcumin

Curcumin (Cur) is a polyphenol isolated from *Curcuma longa*. It exhibits antioxidant and anti-inflammatory effects and has features typical of a neuroprotectant. In 2017, Wang et al. [127] published an analysis of 113 papers on the effects of Cur on PD, of which 13 were identified as significant. Observations were made that Cur exhibits anti-inflammatory, antioxidant, and antiapoptotic effects in the animal model of PD. The anti-inflammatory effect, which is based on the protective effect of Cur against DNA damage and a reduction in cytokine levels, was confirmed in five articles. The ability of Cur to reduce the level of organic radicals, lipid peroxidation, and nitric oxide generation was found in in vivo studies. Due to these properties, Cur meets the definition of a nutraceutical, as it has both nutritional value and the characteristics of a pharmaceutical agent, e.g., leading to the abolition of apoptosis and reduction in oxidative damage in nerve cells. Thus, it could possibly be used as a supportive therapy in the treatment of PD. 

Cur has strong evidence of its ability to modulate autophagy in vivo and in vitro (Table 6). For example, Cur supplementation has been found to induce autophagy in the passive Heymann nephritis rat model [128]. The authors postulated that Cur acts through the PI3K/AKT/mTOR and Nrf2/HO-1 pathways and can significantly alleviate the development of membranous nephropathy. Furthermore, Cur has been reported to reduce the levels of blood glucose, serum creatinine, urea nitrogen, and urine albumen in rats with diabetic nephropathy [129]. On a cellular level, Cur caused an increase in the autophagosome level and upregulation of autophagy-related genes in an animal model and MPC5 cells [129], suggesting possible induction of autophagy. Moreover, Cur has been shown to protect hepatocytes from cobalt chloride-induced epithelial to mesenchymal transition (EMT) by inhibition of the TGF-β/Smad pathway, thus reversing EMT [130]. Furthermore, through inhibition of the TGF-β/Smad pathway, Cur inhibited EMT by activating autophagy via upregulation of the AMPK and PI3K/AKT/mTOR signaling pathways [131].

Epidemiological studies reported an association between carcinogenesis and iron overload [147]. Yang and colleagues [134] presented results from study on castrate-resistant prostate cancer (CRPC) cells, in which they reported that Cur supplementation led to an induction of autophagy and apoptosis in CRPC cells. Additionally, to assess the role of iron, they treated the cells with ferric ammonium citrate (FAC) with or without Cur. Cur was found to bind to FAC in a 1:1 ratio, and further incubation of CRPC cells with Cur and FAC counteracted the Cur-induced autophagy and apoptosis, suggesting the possible mechanism of Cur’s interaction with iron.

Numerous studies have confirmed the protective effect of Cur on neuronal cells. For example, in a study by Rahaman et al. [135], Cur supplementation alleviated arsenic-induced cytotoxicity and reversed the induction of autophagy in PC12 cells. Furthermore, Cur has been shown to inhibit the differentiation of neural stem cells into GFAP^+^ astrocytes or DCX^+^ immature neurons [136]. However, it has also been shown to induce neuronal differentiation of human pluripotent embryonal carcinoma cells and autophagy-mediated neurogenesis, suggesting that its mechanism of action during differentiation is more complex and needs to be further investigated [132]. Lee et al. [133] reported that treatment with Cur caused an induction of autophagy and loss of viability of human glioblastoma A172 cells. This effect was decreased by inhibition of Atg5 and Beclin-1 expression with targeted siRNA. Cur, through inhibition of the PI3K-Akt/mTOR pathway, induces autophagy and inhibits mitophagy in cultured glioblastoma multiforme cells [143]. It has been found to induce autophagy via inhibition of GSK-3β in the human neuroblastoma SH-SY5Y cell line, causing a reduction in amyloid-β precursor protein and α-syn levels through the lysosomal/autophagy pathway [14].

TFEB is being considered as a new possible therapeutic target in the treatment of PD, as it is the master regulator of autophagy processes. A Cur derivative named E4 has been reported to activate TFEB through inhibition of the Akt-mTORC1 pathway, leading to the degradation of α-syn and protection against MPP^+^ toxicity in neuronal cells in vitro [15]. 

Exposure to paraquat (PQ) recently emerged as a risk factor of developing PD. PQ is a neurotoxic agent, whose mechanism of toxicity is complex, although oxidative stress plays an important role in its neurotoxicity [148,149,150]. Cur protected SH-SY5Y cells from PQ-induced toxicity by alleviating ROS generation and apoptotic cell death [137]. Additionally, pretreatment with Cur reversed autophagy induction via downregulation of LC3-I/II expression [137].

A paper by Perrone and colleagues [151] summarizes current knowledge about the potential use of Cur in the treatment of neurodegenerative diseases. Despite the low bioavailability of Cur due to its hydrophobic structure, it is able to restore autophagic flux in dopaminergic neurons, even with A53T mutation of the *SNCA* gene increasing the rate of aggregation of α-syn. The exact mechanism of Cur-induced degradation of protein aggregates in PD is yet to be understood, although autophagy induction seems to be the most plausible explanation.

Based on the critical review by Forouzanfar et al. [152], it can be somewhat concluded that: (1) Cur is more likely to induce autophagy in animal models than in cell cultures [138,139,144,145]; (2) tetrahydroCur is a more potent autophagy inducer than Cur [144,145]; and (3) the response of in vitro models to Cur supplementation often depends on a decrease in LC3-II expression [140,146]. Additionally, Cur has been found to reduce protein aggregates in an in vitro model of PD via a reduction of mTOR/p70S6K signaling and recovery of macroautophagy [141]. Liu et al. [142] reported similar results in which Cur induced autophagy via the AKT/mTOR/p70S6K pathway in ovarian cancer cell lines. This suggests that modulation of the mTOR/p70S6K pathway may not be a neuron-specific mechanism of Cur action.

The Cur mechanism of action seems to be more complex than simple modulation of the antioxidative equilibrium. As shown in Table 5, the response to Cur supplementation varies between the tested models, suggesting the possible selectiveness of its action. The biggest challenge of therapy with Cur is its low bioavailability due to its hydrophilic structure. Despite these drawbacks, Cur could be utilized as a supplement for patients with NDs; however, its structure should be further studied to overcome the bioavailability issue.

### 2.7. Ergothioneine

At physiological pH, ergothioneine (ERG) has a thione structure, making it more stable than thiol antioxidants (it does not undergo autoxidation) [153]. Additionally, ERG has been found to chelate metals [154]. The highest amounts of ERG can be found in mushrooms (up to 1000 times more than in other foods) and fermented foods, such as tempeh. Due to its hydrophilic structure, ERG does not spontaneously penetrate the membrane of cells. Instead, it is actively transported by OCTN1 (organic cation transporter novel type 1). Recently, unification of the abbreviation OCTN1 with SLC22A4 (solute carrier family 22 member 4) or even ETT (ergothioneine transporter) has been suggested because OCTN1 does not mainly transport organic cations as previously thought. ETT is present in virtually all tissues of the human body, including neurons and the blood–brain barrier (BBB). This allows ERG to permeate BBB and reach dopaminergic neurons. For example, ERG supplementation to amyloid-β-treated PC12 cells leads to inhibition of amyloid-induced apoptotic cell death [155]. Moreover, ERG has been found to reduce the antiproliferative effect induced by cisplatin on PC12 cells in vitro and rat neurons in vivo [156]. Rats treated with neurotoxic N-methyl-D-aspartate and supplemented with ERG showed lower neuronal loss when compared to the animals without ERG supplementation [157]. Despite its many advantages, there is no data indicating that ERG can induce or inhibit autophagy in neurons. However, it is indeed a promising nutraceutical that deserves more attention from the scientific community. 

Furthermore, the metabolism of ERG is quite simple. It is absorbed by gastrointestinal epithelial cells and via the blood flow reaches almost every cell. Additionally, ERG significantly accumulates in the liver [158]. The transport of ERG into the cell mainly depends on the activity and expression of ETT. Recently, SLC25A15 and SLC22A5 were also found to transport ERG in vitro; however, their exact role in this process remains unknown [159]. The metabolism of ERG also seems to be relatively slow [160].

Despite the low number of original articles published about ERG, many of them are on the use of ERG in nerve cells, especially in the context of NDs. Indeed, the relevance of ERG in NDs has been the subject of many original and review articles. For example, patients with PD have been shown to have lower serum ERG levels than healthy people, which has been proven at least a few times to date [161,162,163]. Mori et al. [164] conducted a clinical trial (double-blind, simultaneous, placebo-controlled) in which people with diagnosed mild cognitive impairment (MCI) aged 50–80 years were administered an extract of *Hericium erinaceus* mushroom orally in the form of a tablet. It needs to be noted that *H. erinaceus* contains a significant amount of ET [165]. Participants from the study group showed a significantly better performance in cognitive function tests than participants from the placebo group after 16 weeks of therapy. Additionally, a survey of Singaporean residents showed an association between the amount of mushroom consumption and the incidence of age-related conditions, such as dementia or MCI [166], once again suggesting that the mushrooms contain a significant number of compounds that have the ability to slow the deterioration of cognitive functions.

As of early 2022, a total of 689 articles have been published on ERG (according to PubMed). When compared to the other compounds discussed in this section (e.g., Cur = 50,703, vitC = 135,052, carotenoids = 59,430), this represents only a fraction of the knowledge we have about ERG. However, considering its neuroprotective abilities, the presence of ETT in practically every tissue of the human body, and its decent antioxidant properties, one can think that in the near future, it will enter the canon of the most willingly studied substances in the treatment of neurodegenerative diseases. Unfortunately, there are few or even no reports on the potential of ERG to modulate autophagy. There are also no published clinical trials, and that is why such reports are crucial to assess the safety and effects of ERG on human health. However, there are some ongoing clinical trials, such as ISRCTN25890011 led by Dr. Moore from the University of Leeds, assessing the effects of ERG supplementation in people with metabolic syndrome [167]. 

Neuroprotection is often considered the most important feature of ERG in humans. Indeed, the interest around ERG is often focused on its ability to overcome neurotoxicity, usually induced by excess ROS. The role of ERG in the brain has already been reviewed a few times, most recently by Ishimoto and Kato [168] in their review titled “Ergothioneine in the brain”. Besides the excellent description of ERG, they also suggested a role for ERG in certain diseases of the central nervous system. For example, ERG has been found to promote neuronal differentiation via phosphorylation of mTOR and TrkB (tropomyosin receptor kinase B) [169]. Additionally, they highlighted that ETT is—besides the plasma membranes—also expressed in mitochondria [170], and that ERG protects cells from mitochondrial DNA damage induced by hydrogen peroxide [171]. Interestingly, ERG has been proposed as a potential drug for the treatment of depression because of its mTORC1- and TrkB-modulating activity. This hypothesis has been confirmed in mice with depression, in which ERG supplementation significantly improved their health status most likely via the promotion of neurogenesis in the hippocampus [172]. It has been further confirmed in rats, in which ERG ameliorated social defeat stress-induced social avoidance behaviors and sleep abnormalities [173]. 

The concentration of ERG in mushrooms varies between species, and thus it is essential to survey patients on which particular species is part of their diet. ERG seems to be a perfect candidate for various applications that are not only medicine related. For example, it has been proposed as a food additive, preventing the oxidation and discoloration of beef and fish meat, meaning that it could be used to keep food fresh and good-looking for a longer time [174], especially since ERG is a colorless substance with a slightly sweet taste [Lalitphan Kitsanayanyong unpublished data]. These properties give ERG an advantage over mushroom extract (ME), which has a more or less mushroom taste and brownish color. Because of this, ME cannot be used in every food product (especially in those that have a delicate taste and bright colors), which is why ERG could become a perfect alternative. Beelman et al. [175] compared the consumption of ERG-rich mushrooms with the AD and PD death rate in some countries and suggested that there is a connection between ERG consumption and the mortality rate from NDs. They also calculated—based on available data—that the average consumption of ERG/day in Italy is 4-fold higher (4.6 mg/day) than in the USA (1.1 mg/day), which correlates with the AD/PD death rate in those countries (Italy has a significantly lower death rate due to NDs). Furthermore, higher ERG consumption contributes to lower overall mortality and longer life expectancy [176]. ERG has been even been called the “longevity vitamin” [176], which is fully understandable based on the available data, although there is still much to discover. Hopefully, ERG will be given more attention in the coming years.

### 2.8. Lipoic Acid

Lipoic acid (LA) is a carboxylic acid that is endogenously synthesized in humans and contained in some foods. It mainly functions as an antioxidant and cofactor of several enzymes. Zhang et al. [177] verified its antioxidant and anti-inflammatory properties against neurons in vivo in a mouse model of PD. LA supplementation (31.5 or 63 mg/kg) during L-dopa therapy reduced the incidence of L-dopa-induced dyskinesia in a dose-dependent manner without affecting the therapeutic effect of the drug. In addition, LA supplementation decreased malondialdehyde levels and increased GSH activity. Li et al. [178] administered LA (100 mg/kg) to mice with LPS-induced PD and reported an improvement in motor function, reduction in α-syn accumulation, and activation of proinflammatory markers after treatment with LA. Of particular interest is the reduction in the accumulation of protein deposits, as this can be considered twofold. LA could, through its antioxidant activity, inhibit processes leading to the accumulation of protein oligomers or induce the degradation of protein deposits, such as the process of autophagy. Nevertheless, the use of LA in combination with a drug could bring about the expected effects in PD patients; however, this requires further mechanistic studies. In Table 7 some of the available data on the modulation of autophagy by LA have been summarized.

α-Lipoic acid (α-LA) has been found to attenuate renal stress by inhibiting autophagy and apoptosis in human proximal tubular epithelial cells [179]. The MAPK/NF-κB signaling pathway-modulating activity of α-LA has been suggested as a mechanism of its action in proximal tubular epithelial cells. Furthermore, α-LA has been shown to protect the hepatic stellate cells of rats against thioacetamide-induced liver fibrosis by downregulating the expression of autophagy-specific genes [180]. Additionally, α-LA has been found to inhibit O^6^-methylguanine-DNA methyltransferase (MGMT), a protein responsible for removing DNA adduct O^6^-methylguanine, which is highly cytotoxic for cancer cells, with a simultaneous induction of autophagy in a Beclin-1-dependent manner [181]. 

The role of autophagy is often studied during myocardial ischemia/reperfusion injury (I/RI). α-LA has been shown to downregulate the I/RI-mediated induction of autophagy, contributing to enhanced cell survival and decreased cell loss in H9c2 cardiomyocytes [182]. Similar results were reported by Qiu et al. [183] in vascular smooth muscle cells isolated from rats with T2DM, in which LA protected the vascular functions via downregulation of autophagy. However, LA has also been found to induce autophagy in the myocardium, kidney, and small intestine of septic rats, protecting the organs from excess apoptosis and cell loss, which is in contrast with previously presented results [184].

### 2.9. N-Acetylcysteine

Extensively detailed and willingly used in in vitro studies, N-acetylcysteine (NAC) functions as an antioxidant and precursor of glutathione (GSH). NAC supplementation in vivo has been shown to increase the activity of complex I and IV of the oxidative phosphorylation chain and reduce mitochondrial damage due to ROS accumulation, consequently leading to protection against dopamine-induced neuronal death [186]. Intravenous administration of NAC caused an increase in the GSH level in the brain and blood of PD patients [187]. NAC protects nerve cells in vitro from rotenone toxicity and leads to the activation of dopamine transporter in vivo [188]. Due to these properties, it could potentially be used alongside L-dopa to protect nerve cells from persistent oxidative stress. However, there are no clinical studies of simultaneous therapy of both NAC and L-dopa.

Some of the available papers reporting the ability of NAC to modulate the autophagy process are summarized in Table 8. To start with, NAC supplementation has been found to alleviate paraquat-induced oxidative stress in primary murine neural progenitor cells, resulting in the suppression of autophagy and partial inhibition of *Pink1* and *Parkin*-mediated mitophagy [189]. Another study reported that NAC attenuated olanzapine-induced overexpression of the LC3-II protein in mHypoA-59 hypothalamic neurons [190]. Additionally, NAC-mediated overexpression of autophagy markers has been reported in rats after abdominal aortic constriction operation [191]. 

β-Conglycinin is a major allergen in soybeans, inducing intestinal dysfunction and diarrhea in human infants. Wang and colleagues [192] reported that treatment with NAC reduced the incidence of diarrhea and downregulated autophagy markers in the jejunum of 12-day-old piglets challenged with β-conglycinin. NAC is also often used as an ROS-protecting agent. However, as reported by Kim et al. [193], NAC was not able to attenuate the effects of radiation-induced oral mucositis in rats. Further histopathologic analysis revealed that NAC treatment significantly reduced the level of LC3 protein and ROS generation. They also tested the effect of NAC supplementation in vitro on keratinocytes, which caused a significant reduction in autophagy markers. NAC has also been found to reverse antiretroviral therapy-induced autophagy in primary rat microglial cells. For this reason, it has been suggested as a potentially relevant supportive therapy [194]. 

Myocardial ischemia reperfusion injury (I/RI) is one of the complications that can develop as a consequence of diabetes. It is thought that I/RI is caused by excess oxidative stress and autophagy. Wang and colleagues [195] presented data from an in vivo study on rats with STZ-induced diabetes that were supplemented with NAC before being subjected to coronary occlusion and reperfusion. The results showed that NAC significantly alleviated I-RI-induced oxidative stress, apoptosis, and autophagy [195].

It seems that, generally, NAC exerts its autophagy-modulating activity via a reduction in oxidative stress. The papers summarized in Table 7 suggest that when used at a concentration higher than physiological, NAC can protect cells from excessive autophagy. However, more specific data is needed to allow a conclusion on its relevance in the treatment or prevention of PD and other synucleinopathies to be drawn.

### 2.10. Polyunsaturated Fatty Acids (PUFAs)

Fish and fish oils, walnuts, and edible seeds are the main sources of omega-3 fatty acids in the human diet. The healthy effects of PUFAs (polyunsaturated fatty acids) result from the presence of polyunsaturated carbon chains in their structure. PUFAs have been shown to protect nerve cells [196]. Taghizadeh et al. [197] presented the results from a clinical study on PUFAs and vitE supplementation of PD patients (randomized, double-blind, placebo-controlled) that were given omega-3 fatty acids (1000 mg) along with vitE (400 IU) daily for 3 months. The results indicated that such supplementation significantly improved the health status of the patients. A decrease in hs-CRP levels and an increase in the total blood antioxidant capacity were observed. These results suggest that supplementation with PUFAs and vitE in PD patients may result in an improvement in the clinical symptoms of the disease and the blood oxidative equilibrium.

There is no doubt that PUFAs present many advantages for human health. They are mostly known to have antioxidative and anti-inflammatory effects. Reports that PUFAs modulate autophagy in various conditions have been published as well. Some of them are summarized in Table 9. For example, ω-3 PUFA supplementation of rats with traumatic brain injury (TBI) protected neurons via upregulation of SIRT1-mediated deacetylation of Beclin-1 against TBI-induced apoptotic death [198].

ω-6 PUFAs have been distinguished as essential fatty acids in the process of macroautophagy [199]. Similarly, ω-3 PUFAs (docosahexenoic acid (DHA) and eicosapentaenoic acid (EPA)) reduce lipid accumulation and inhibit apoptosis in a free fatty acid-induced NAFLD model in vitro via induction of autophagy by downregulation of Stearoyl-CoA Desaturase1 expression in hepatocytes [200].

*Fat-1* transgenic mice are capable of self-synthesizing ω-3 PUFAs, and thus they are the perfect model to study the effect of PUFAs on a range of conditions. For example, Hwang et al. [201] reported that *fat-1* transgenic modification protected mice against STZ-induced β cell death by activating autophagy in β cells. As reported by Fang et al. [202], the *fat-1* mice had a lower overall body weight and body length than wild-type (WT) mice. Additionally, *fat-1* mice were reported to have a higher basal level of autophagosomes in the arcuate nucleus, downregulated p62 and upregulated Atg7 protein expression, and an increased LC3-II/LC3-I ratio compared to WT mice [202]. Additionally, these mice were less susceptible to STZ-induced cell damage, and did not develop hyperglycemia, motor deficits, or Purkinje cell loss when compared to WT mice [203]. Endogenous ω-3 PUFAs in *fat-1* mice with concanavalin A-induced hepatitis were shown to protect the liver of the mice from damage, which was evidenced by decreased mortality, attenuated hepatic necrosis, lessened serum ALAT activity, and inhibited production of proinflammatory cytokines [204]. Further studies revealed that endogenous ω-3 PUFAs significantly increased the autophagy markers in hepatic T cells of *fat-1* mice after concanavalin A treatment when compared to WT mice, suggesting that the protective effect of PUFAs may depend on the activation of the autophagy process [204].

Autophagy is being considered as one of the main processes involved in the repair of renal ischemia-reperfusion injury. Indeed, *fat-1* mice have better renal cell survival and overall renal function, and express less pathological renal damage than WT mice after I-RI [205]. Additionally, *fat-1* mice are better protected against excess oxidative stress [205]. The induction of autophagy via the activation of AMPK by ω-3 PUFAs was also confirmed by Choi et al. [206] during *Toxoplasma gondii* infection of bone marrow-derived macrophages isolated from *fat-1* mice. They identified ω-3 PUFAs as being important during *T. gondii* infection.

Glioblastoma cell lines supplemented with DHA exhibit an upregulation of the LC3-II protein level, autophagic flux activation with AMPK activation, and decreased mTOR activity [207]. Analysis of glioblastoma tumor tissue from *fat-1* mice revealed that supplementation with DHA caused a decrease in the p-Akt level accompanied by an increase in the p-AMPK level [207]. 

α-syn contains a lipid-binding motif at the N-terminal site of its structure, suggesting possible interactions with lipid elements of the cell’s membrane [208]. Moreover, α-syn shows a high affinity for PUFAs, and as reported by Davidson et al. [209], and lipid-bound α-syn has approximately 80 % higher alpha-helicity. Therefore, the interaction with lipids stabilizes its α-helical structure. Lipid peroxidation of PUFAs, such as DHA and arachidonic acid (ARA), generates reactive aldehydes, of which 4-oxo-2-nonenal and 4-hydroxy-2-nonenal were found to modify α-syn and produce oligomers of this protein in vitro [210,211]. In contrast with native α-syn, these aldehyde-mediated oligomers were found to be toxic to SH-SY5Y cells [212]. However, PUFAs were found to upregulate autophagy (Table 8), which is essential during the clearance of α-syn in neurons. Indeed, it is possible that lipid peroxidation of PUFAs generates aldehydes that were reported to react with α-syn, producing its oligomers in vitro [210,211], although the PUFA-mediated upregulation of autophagy leads to rapid degradation of these oligomers before they become cytotoxic to the cells. Despite the numerous papers published on this topic, this issue requires further research.

## 3. Conclusions

The importance of autophagy during PD is a well-known fact. New experimental therapies for PD are often focused on restoring autophagic flux. However, these therapies often cause a number of side effects, which are especially troublesome for the elderly. On the whole, this is where nutraceuticals could be utilized to overcome drug-induced side effects, improve the conventional therapy, and increase the adherence to therapy.

Besides the compounds that were described in this review, there are many other promising substances that exhibit autophagy-modulating activity, and thus could be utilized in the treatment of PD. For example, ursolic acid (UA) is a pentacyclic triterpenoid with antioxidative and anti-inflammatory abilities that has been thought to be pharmacologically inactive for a long time [213]. However, UA has been found to be neuroprotective against MPTP-induced lipid peroxidation and inflammation in PC12 cells [214]. In their review, Ramos-Hryb et al. [215] described the relevance of UA in the treatment of NDs and psychiatric diseases. Conclusions were made that the available data supports the beneficial role of UA in NDs and psychiatric disorders. However, there is only a little clinical data on the use of UA, and thus its impact on human health remains unclear [215].

Furthermore, chlorogenic acid (CGA) is a compound found in, for example, coffee, tea, and many edible plants [216]. Interestingly, caffeinated coffee contains seven to nine times more CGA than caffeine [217]. It is worth mentioning that commercially, CGA is mostly available in the form of 5- O-caffeoylquinic acid, which can pass through BBB in its pure form or as a metabolite [218]. The neuroprotective abilities of CGA have already been confirmed in vitro. CGA has been reported to attenuate MPTP-induced neuroinflammation in SN via regulation of NF-κB expression in a mice model of PD [219]. Furthermore, CGA rescued PC12 cells from α-syn-induced and oxidized dopamine cytotoxicity in a dose-dependent manner [218]. More data were analyzed by Heitman and Ingram [216] in their review about CGA, in which they concluded that it is an antioxidant with promising potential for neuroprotection. It is, indeed, a compound that deserves more attention and a clinical approach.

**Figure 2:**
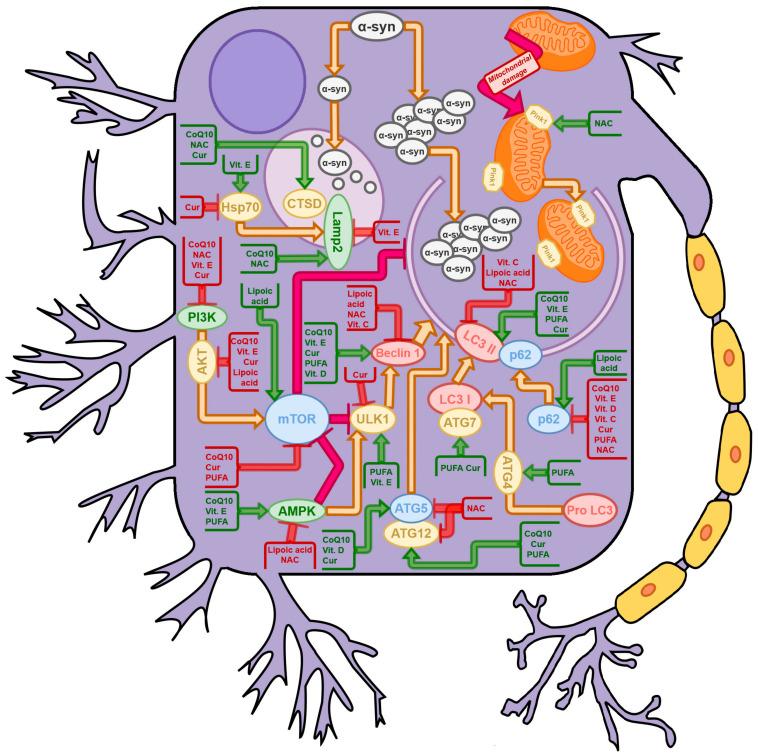
Diagram presenting the modulation of the autophagy pathway in mammalian nerve cells induced by compounds described in this review. The green arrows indicate induction of mRNA/protein expression, whereas the red arrows indicate an inhibitory effect of the presented compounds.

Figure 2 presents the effects induced in neuronal cells by the compounds listed in this review. For example, supplementation with curcumin could inhibit CMA through inhibition of Hsp70 and in turn induce MA via induction of Atg5, Atg7, and Atg12 protein expression. These interactions are certainly complex and can vary depending on the experimental model and conditions. Hence, it is crucial to assess the impact of a combination of multiple compounds, especially those that were proven to modulate the autophagy process in PD.

Research suggests that the presented nutraceuticals can help in the management of autophagy during synucleinopathies. Nutraceuticals are usually well-tolerated and cause little or no side effects. For this reason, they could be utilized in the support of conventional therapy of Parkinson’s disease. We believe that our review will contribute to the understanding of the importance of nutraceuticals and will shed some light on their autophagy-modulating activity.

## Figures and Tables

**Table 1:** Summary of selected available reports on the modulation of autophagy with vitamin A and its derivatives.

Compound Used	Experimental Model	Signaling Mediators	Results	Ref.
ATRA	SKBR3 and MDA-MB453 breast cancer cell lines	↑LC3-II/I (SKBR3)	ATRA-induced autophagy was mediated by RARα in SKBR3 cells.	[49]
Crocin	Middle cerebral artery occlusion rat model	↑p62 ↑p-mTOR/mTOR ↓LC3-II/I ↓p-AMPK/AMPK ↓ULK1	Crocin reduced the level of autophagy following cerebral ischemia by activating mTOR.	[50]
β-carotene	Rats with LPS-induced intestinal inflammation	↑p-AKT/AKT ↓LC3-II/I	β-carotene protected rat intestinal cells, most probably via the JAK2/STAT3 and JNK/p38 MAPK signaling pathways.	[51]
Lycopene (Lyc)	Cadmium-induced hippocampal dysfunction mice	↓Beclin-1 ↓AKT1 ↓MAPK ↓Atg * mRNA	Lyc reversed Cd-induced dysfunctions and neurotoxicity.	[52]
	Endothelial progenitor cells isolated from T2DM rats	↓Beclin-1 ↓LC3-II/I	Lyc promoted EPCs survival and protected EPCs from apoptosis and autophagy induced by AGEs.	[53]
	Gentamicin-induced renal cortical oxidative stress rat model	↓LC3-II/I	Lyc decreased the level of the LC3-II/I autophagy marker.	[54]
4HPR	hiPSC-derived motoneurons from ALS patients’ keratinocytes	↑LC3-II/I ↑SQSTM1 ↓ATG10	4HPR induced autophagy and downregulated Atg10 expression via modulation of TBK1.	[55]
Astaxanthin	*H. pylori*-infected AGS cells	↑LC3-II/I ↑p-AMPK/AMPK ↑p-ULK1/ULK1 ↓p62 ↓p-mTOR/mTOR	Astaxanthin increased autophagy through activation of the AMPK pathway.	[56]
Lutein	Cobalt (II) Chloride-Induced Hypoxia in Rat-derived Müller Cells	↓LC3-II/I	Lutein suppressed autophagosome formation after hypoxic insult and inhibited autophagy after rapamycin treatment.	[57]
	IEC-6 rat intestinal epithelial cells	↑LC3-II/I ↑Beclin-1 ↑p-AMPK ↑p-JNK ↑p-p38 ↑p-mTOR	Lutein induced autophagy via the upregulation of Beclin-1 in IEC-6 cells.	[58]
	ICG and BBG-treated ARPE-19 and 661W cell lines	↑LC3-II/I	Lutein induced autophagy and diminished the cytotoxic effects of ICG and BBG in ocular cells.	[59]
Fucoxanthin	Nasopharyngeal carcinoma C666-1 cell line	↑LC3-II/I ↑p62 ↑ATG7 ↓ATG4B	Fucoxanthin induced autophagy and apoptosis in C666-1 cells.	[60]
	Traumatic brain injury (TBI) mice model	↑LC3-II/I ↑Beclin-1	Fucoxanthin exerted protective effects, potentially via regulation of the Nrf2-autophagy pathway.	[61]

Abbreviations: ATRA—all-trans retinoic acid; RARα—pan-retinoic acid receptor; LPS—lipopolysaccharide; AGE—advanced glycation end products; RA—retinoic acid; T2DM—type 2 diabetes mellitus; EPC—endothelial progenitor cells; hiPSC—human-induced pluripotent stem cell; ALS—amyotrophic lateral sclerosis; 4HPR—4-hydroxy(phenyl)retinamide; TBK1—TANK-binding kinase 1; ARPE-19—human retinal pigment epithelial cells; 661W—mouse photoreceptor cells; ICG—indocyanine green; BBG—brilliant blue G; AGS—human gastric adenocarcinoma cell line; ARPE-19—spontaneously arising retinal pigment epithelia cell line; 661W—murine cone photoreceptor cell line; C666-1—Epstein–Barr virus-infected undifferentiated nasopharyngeal carcinoma cell line; * Included: ATG2b, ATG3, ATG4B, ATG5, ATG7, ATG9A, ATG9B, ATG13, ATG14, ATG16-2.

**Table 2:** Summary of available reports on the modulation of autophagy by vitamin C and its derivatives.

Compound Used	Experimental Model	Signaling Mediators	Results	Ref.
AA	Pilocarpine-induced rat model of seizures	↓Beclin-1	AA partially inhibited the pilocarpine-mediated induction of oxidative stress and autophagy.	[73]
	Murine bone marrow stromal cells (BMSCs)	↓LC3 ↓p62	VitC significantly rescued BMSCs from oxidative stress by regulating autophagy.	[74]
SA	Methamphetamine-treated primary rat cortical neuron-glia cells	↓LC3-II/I ↓Beclin-1	VitC partially attenuated the induction of autophagy, most probably via an ROS-dependent mechanism.	[75]

Abbreviations: AA—ascorbic acid; SA—sodium L-ascorbate.

**Table 3:** Summary of selected available reports on the modulation of autophagy by vitamin D and its derivatives.

Compound Used	Experimental Model	Signaling Mediators	Results	Ref.
1,25(OH)_2_D_3_	SiO_2_-mediated lung injury in vitro model (THP-1 and BEAS-2B cells)	↑LC3 ↓p62	VitD protected against particle-induced cell damage via the induction of autophagy in an Nrf2-dependent manner.	[79]
	*P. gingivalis*-infected U937-derived macrophages	↑LC3-II/I ↑Atg5 ↓p62	VitD induced autophagy to degrade live *P. gingivalis.*	[80]
	STZ-induced T2DM mouse model	↑Beclin-1 mRNA ↑LC3 mRNA	VitD induced autophagy and suppressed apoptosis of pancreatic β cells.	[81]
	MCF-7 cell line (with or without VDR knockout)	↑LC3	VitD increased the level of autophagy in MCF-7 cells. VDR gene knockout caused even higher than vitD upregulation of autophagy, suggesting a possible VDR-dependent mechanism of autophagy modulation.	[82]
25(OH)D_3_	UV-mediated acute skin injury mouse model	↑LC3-II/I ↓p62	VitD resolved skin injury via inhibition of inflammatory cytokines associated with enhanced autophagy in myeloid anti-inflammatory M2 macrophages.	[83]
VitD_3_	*Aspergillus fumigatus*-infected mice model	↓LC3-II	VitD delayed the formation of lysosomes against *A. fumigatus* through autophagy.	[84]
VitD deficiency	Two groups of patients: HCV HCV + HCC	↓LC3	Downregulation of autophagy was observed in vitD_3_-deficient patients.	[85]

Abbreviations: 1,25(OH)_2_D_3_- 1,25-dihydroxycholecalciferol; VitD_3_—cholecalciferol; HCC—hepatocellular carcinoma; HCV—hepatitis C virus; 25(OH)D_3_—25-hydroxycholecalciferol; VDR—vitamin D receptor; KO—knockout; THP-1—human acute monocytic leukemia cell line; BEAS-2B—human bronchial epithelium cell line transformed with Ad12-SV40 2B; U937—human adult acute monocytic leukemia cell line; VDR—vitamin D receptor; MCF-7—triple-positive human breast cancer cell line; HCV—hepatitis C virus.

**Table 4:** Summary of selected available reports on the modulation of autophagy by vitamin E and its derivatives.

Compound Used	Experimental Model	Signaling Mediators	Results	Ref.
VitE deficiency	Hippocampal neurons isolated from vitE-deficient mice	↑LC3-II	VitE deficiency led to axonal degeneration. LC3-II expression is higher in short-term rather than long-term deficiency.	[94]
α-tocph	Rats with pilocarpine-induced status epilepticus	↓LC3-II/I ↓Beclin-1	α-tocph inhibited autophagy in the hippocampus of the animals.	[95]
		↓Lamp2a	α-tocph inhibited CMA in the hippocampus of rats.	[96]
	Proximal tubules isolated from a DN patients/rat model	↓LC3-II ↓SQSTM1	High dose of α-tocph downregulated the autophagy markers.	[97]
	Chronic unpredictable mild stress mice	↑LC3-II ↑p-AMPK/AMPK ↑ULK1Ser317 ↓p62 ↓p-mTOR/mTOR ↓p-P70S6K1	α-tocph induced antidepressive responses via the promotion of autophagy in chronic unpredictable mild stress mice.	[98]
	Primary rat hepatocytes	↑LC3-II/I	Both compounds increased autophagy by accelerating LC3 conversion.	[99]
α-toctr	H-4-II-E cells			[85]
γ-toctr	Ischemia/reperfusion rat model	↑p-Akt/Akt ↑LC3-II/I ↑Beclin-1 ↓p-mTOR/mTOR	γ-toctr-mediated cardioprotection was achieved by its ability to induce autophagy.	[100]
	Human prostate cancer cell lines, PC-3 and LNCaP cells	↑LC3-II/I	γ-toctr promoted autophagy in PC-3 and LNCaP cells.	[101]
	HCT-116 cells	↑LC3-II/I	γ-toctr induced autophagy.	[102]
	Mouse (+SA) and human (MCF-7) mammary cancer cells	↑LC3-II/I ↑Beclin-1 ↓PI3K ↓p-Akt ↓p-mTOR	γ-toctr induced autophagy in cancer (+SA, MCF-7) cell lines but did not in normal mammary cell lines (CL-S1, MCF-10A).	[103]
Palm oil TRF	Rat pancreatic stellate cells	↑LC3-II/I	TRF reduced the viability of activated PSCs by targeting the mitochondrial permeability transition pore.	[104]
α-TEA	4T1 and 3LL cells	↑LC3-II/I	Autophagy and apoptosis signaling pathways are activated during α-TEA-induced death of cells.	[105]

Abbreviations: CMA—chaperone-mediated autophagy; DN—diabetic nephropathy; H-4-II-E—rat hepatoma cell line; PC-3—human grade IV prostate cancer cell line; LNCaP—androgen-sensitive human prostate adenocarcinoma cell line; TRF—tocotrienol-rich fraction; PSC—pancreatic stellate cells; α-TEA—alpha-tocopheryloxyacetic acid; 4T1—highly metastatic tumor cell line; 3LL—Lewis lung carcinoma; γ-toctr—γ-tocotrienol; α-toctr—α-tocotrienol.

**Table 5:** Summary of selected available reports on the modulation of autophagy by coenzyme Q10 and its derivatives.

Compound Used	Experimental Model	Signaling Mediators	Results	Ref.
CoQ10	Methotrexate-induced lung and liver fibrosis rat model	↑LC3 ↑p62 ↓mTOR	CoQ protected against lung and liver fibrosis via induction of autophagy.	[113]
	BPA-treated C2C12 cells	↑LC3-II ↑Lamp2 ↓p62	CoQ promoted autophagy by improving lysosomal function.	[114]
	Fibroblasts derived from an MERRF patient	↑phospho-AMPK ↓LC3-II ↓p62	CoQ restored the autophagic flux in MERRF fibroblasts.	[115]
	Acetaminophen-induced liver injury mice model	↑LC3-II ↑Parkin ↑mito-p62 ↓p62	CoQ activated mitophagy and protected against acetaminophen-induced liver injury.	[116]
	Heat-stressed chicken primary myocardial cells	↑LC3 ↑Beclin-1 ↑Atg5 ↓p-Akt/Akt ↓p-mTOR/mTOR ↓p-PI3K/PI3K	CoQ protected the cells during heat stress by upregulation of autophagy via the PI3K/Akt/mTOR pathway.	[117]
	Fibroblasts from patients with MELAS	↓LC3-II ↓Beclin-1 ↓Atg12	CoQ partially alleviated MELAS-induced activation of autophagy.	[119]
	NRTI-treated HUVEC cells	↓LC3-II	CoQ prevented an NRTI-mediated increase in LC3-II.	[120]
	Acute myocardial ischemia-reperfusion rat model	↑LC3-II ↑Beclin-1 ↑Atg5 ↓p62	CoQ protected against acute myocardial ischemia-reperfusion injury via the autophagy pathway.	[118]
	Primary pancreatic stellate cells isolated from a mice model of pancreatic fibrosis	↑p-Akt ↑p-mTOR ↑p-PI3K ↓LC3-II/I ↓Atg5 ↓Beclin-1 ↓p62	CoQ alleviated pancreatic fibrosis by the ROS-triggered PI3K/Akt/mTOR pathway.	[121,122]
MitoQ	Sepsis-induced acute lung injury rat model	↑p-Akt/Akt ↑p-mTOR/mTOR ↑p-GSK-3β/GSK-3β ↓Beclin-1 ↓LC3-II/I	MitoQ protected sepsis-induced acute lung injury by activating the PI3K/Akt/GSK-3β/mTOR pathway.	[123]
CoQ deficiency	Fibroblasts from patients with a CoQ deficiency	↑Atg12 ↑Beclin ↑LC3 ↑cathepsin D	Authors suggested a protective role of autophagy in CoQ deficiency.	[124]
Idabenone	SH-SY5Y-A53T cells	↑LC3-II	Idabenone enhanced the autophagy-mediated clearance of α-syn.	[125]
Antroquinonol	PANC-1 and AsPC-1 cells	↑LC3-II ↓p-AKT ↓p-mTOR	Antroquinonol induced anticancer activity through an inhibitory effect on PI3K/Akt/mTOR pathways.	[126]

Abbreviations: BPA—bisphenol A; C2C12—murine myoblast cell line; MERRF—myoclonic epilepsy with ragged-red fibers; MitoQ—mitochondrial coenzyme Q10; SH-SY5Y-A53T—human neuroblastoma cell line with A53T missense point mutation in the SNCA gene; MELAS—mitochondrial encephalomyopathy, lactic acidosis, and stroke-like episodes; NRTI—nucleoside reverse transcriptase inhibitors; HUVEC—human umbilical vein endothelial cells; PANC-1—human pancreatic cancer cell line; AsPC1—human pancreatic ductal metastasis adenocarcinoma.

**Table 6:** Summary of selected available data on the modulation of autophagy by curcumin and its derivatives.

Compound Used	Experimental Model	Signaling Mediators	Results	Ref.
Cur	SH-SY5Y neuroblastoma cells	↑LAMP1 ↑LC3-II ↓p62	Cur regulated autophagy by controlling TFEB through the inhibition of GSK-3β.	[14]
	NTERA2 stem cells	↑LC3 ↑LAMP1 ↑Atg12 ↑Atg5	Cur induced neurogenesis of NTERA2 cells via the activation of ROS-mediated autophagy.	[132]
	Passive Heymann nephritis rat model	↑LC3-II/I ↑Beclin-1 ↓PI3K ↓p-mTOR	Cur induced autophagy through the PI3K/AKT/mTOR and Nrf2/HO-1 pathways.	[128]
	A172 glioblastoma cells	↑LC3-II ↑Atg5 ↑Atg7 ↑Atg12 ↑Beclin-1	Cur induced autophagy and led to the death of cells.	[133]
	CRPC cells (DU145 and PC3 cell lines)	↑LC3-II	Cur induced apoptosis and protective autophagy in CRPC cells.	[134]
	Arsenic-treated PC12 cells	↑mTOR ↑Akt ↑ERK ↑Nrf2 ↓ULK ↓LC3-II	Cur alleviated arsenic-triggered toxicity in PC12 cells by regulating autophagy and apoptosis.	[135]
	Rat neural stem cells differentiated into GFAP^+^ astrocytes or dcX^+^ immature neurons	↓Atg7 ↓p62 ↓ULK	Cur inhibited NSC differentiation into GFAP+ astrocytes or dcX+ immature neurons.	[136]
	Renal tissue derived from an STZ-induced diabetic nephropathy rat model	↑LC3-II/I ↓p62 ↓p-mTOR ↓p-Akt ↓PI3K	Cur protected podocytes by alleviating EMT via the PI3K/Akt/mTOR pathway.	[129]
	SH-SY5Y cells treated with paraquat	↓LC3-II	Cur reversed the paraquat-mediated induction of autophagy in SH-SY5Y cells.	[137]
	Rat model of sciatic nerve injury	↑LC3-II/I ↑Beclin-1 ↑p-Erk1/2 ↓p62 ↓p-Akt	Cur promoted injury-induced cell autophagy, remyelination, and axon regeneration in the sciatic nerve of rats.	[138]
	Double-transgenic mice (hAPP and mhPS1)	↑LC3-II/I ↑Beclin-1 ↓PI3K ↓p-Akt ↓p-mTOR	Cur inhibited Aβ generation and induced autophagy by downregulation of the PI3K/Akt/mTOR pathway.	[139]
	OGD/R model of the PC12 cell line	↑p62 ↓LC3-II/I	Cur exerted neuroprotection via regulation of the reciprocal function between autophagy and HIF-1α.	[140]
	SH-SY5Y neuroblastoma cells with A53T mutation in the SNCA gene	↑LC3-II/I ↓p-mTOR ↓α-syn	Cur efficiently reduced the accumulation of A53T α-synuclein through downregulation of the mTOR/p70S6K signaling and recovery of macroautophagy.	[141]
	SK-OV-3, A2780 cell lines	↑LC3-II/I ↓p-Akt ↓p-mTOR ↓p-p70S6K	Cur induced protective autophagy via inhibition of the AKT/mTOR/p70S6K pathway.	[142]
	Hepatic fibrosis rat model	↑Atg-7 ↑Beclin-1 ↑LC3-II/I ↓PI3K mRNA ↓mTOR mRNA ↓SQSTM1 mRNA	Cur effectively reduced the occurrence of EMT via the activation of autophagy.	[131]
Cur and SLCP	U-87MG, GL261, F98 cell lines	↑Atg5 (U-87MG) ↑Atg7 (U-87MG and GL261) ↑Beclin-1 ↑LC3-II/I ↑p62 ↓mTOR ↓PI3K ↓Akt	Increased levels of autophagy and decreased levels of mitophagy markers, along with inhibition of the PI3K-Akt/mTOR pathway were noted. The effects were greater in the SLCP-treated group when compared to Cur.	[143]
	C6-glioma and N2a cell lines	↑Beclin-1 (C6-glioma) ↑p-Akt (C6-glioma) ↓Atg7		
“E4” Cur derivative [144]	N2a cell line	↑LC3-II ↑LAMP1 ↑CSTD ↓p-Akt ↓p-mTOR	E4 induced TFEB activation mainly through Akt-mTORC1 inhibition, promoting the degradation of α-syn, and protected against the cytotoxicity of MPP^+^.	[15]
THCu	Traumatic brain injury (TBI) rat model	↑LC3-II/I ↑Beclin-1	Treatment with THCu improved neurological function via the activation of autophagy and attenuation of oxidative stress.	[145]
		↑LC3-II/I ↑Beclin-1 ↓p62	THCu protected neurons from TBI-induced apoptotic neuronal death.	[144]
AO-2	OGD/R model of primary culture of rat cortical neurons	↑p-Akt ↑p-mTOR ↓LC3-II/I	AO-2 increased the resistance of cortical neurons to OGD/R by decreasing autophagy and cell apoptosis, which involves an mTOR-dependent mechanism.	[146]

Abbreviations: NTERA—embryonal carcinoma stem cells; CRPC—castration-resistant prostate cancer cells; NSC—neural stem cells; SLCP—solid lipid cur particles; GBM—glioblastoma multiforme; hAPP—human amyloid precursor protein (HuAPP695swe); mhPS1—mutant human presenilin 1 (PS1-dE9); THCu—tetrahydroCur; OGD/R—oxygen glucose deprivation/reperfusion; AO-2—7-(4-Hydroxy-3-methoxyphenyl)-1-phenyl-4E-hepten-3-one; BNL CL2—mouse embryonic hepatocytes in logarithmic growth phase; STZ—streptozotocin.

**Table 7:** Summary of selected available data on the LA-modulating activity of autophagy.

Compound Used	Experimental Model	Signaling Mediators	Results	Ref.
α-LA	p-Cresyl sulfate-induced renal tubular injury HK-2 cells	↓LC3-II/I ↓Beclin-1 ↓p-ERK ↓p-p38 ↓p-JNK	LA treatment reduced apoptosis and autophagy by modulating the ER stress and MAPK/NF-κB signaling pathways.	[179]
	TAA-induced liver fibrosis rat model	↓LC3-II/I	LA inhibited autophagy and induced apoptotic clearance of activated HSCs.	[180]
	Colorectal cancer cell lines: HCT116, RKO	↑LC3B ↓p-Akt (HCT116)	LA inhibited MGMT and induced autophagy.	[181]
	H9c2 cardiomyocytes derived from rat myocardium under H/RI	↓Beclin-1 ↓LC3-II/I	Pretreatment with LA inhibited the degree of autophagy and increased the viability of cells.	[182]
	Vascular smooth muscle cells isolated from rats with STZ-induced T2DM	↑p62 ↑p-mTOR ↓Beclin-1 ↓p-AMPK	LA treatment reduced the autophagy-related index and activation of the AMPK/mTOR pathway in an H_2_S-dependent manner.	[183]
	Heart, kidney, and small intestine cells isolated from rats with sepsis	↑LC3-II/I ↑Atg5 ↑Atg7 ↑Beclin-1 ↓p62	LA upregulated autophagy in the myocardium, kidney, and small intestine of septic rats and reduced apoptosis.	[184]
	3T3-L1 preadipocytes during adipogenesis	↑p-mTOR ↑p62 ↓p-AMPK ↓LC3-II/I	LA significantly attenuated adipocyte differentiation and consequently decreased the intracellular fat deposit of adipocytes.	[185]

Abbreviations: α-LA—alpha-lipoic acid; HK-2—human renal proximal tubular epithelial cells; TAA—thioacetamide; HSCs—hepatic stellate cells; HCT116—human epithelial colorectal carcinoma cell line; RKO—human epithelial colon cancer cell line; MGMT—O^6^-methylguanine-DNA methyltransferase; H/RI—hypoxia/reoxygenation injury; T2DM—type 2 diabetes mellitus.

**Table 8:** Summary of data on the modulation of autophagy by N-acetylcysteine.

Compound Used	Experimental Model	Signaling Mediators	Results	Ref.
NAC	PQ-treated primary murine neural progenitor cells	↑mTOR ↓LC3B ↓Pink1/Parkin	NAC alleviated PQ-induced cytotoxicity and reversed the induction of autophagy.	[189]
	Abdominal aortic constriction rat model	↓LC3B ↓Beclin-1 ↓Atg12 ↓p-PI3K/PI3K	NAC reversed the AAC-induced activation of autophagy.	[191]
	Olanzapine-treated mHypoA-59 cells	↓LC3-II	NAC mitigated the olanzapine-induced upregulation of LC3-II.	[190]
	Piglets challenged with β-conglycinin	↑Beclin-1 ↑LC3B-I ↓Atg5	NAC supplementation improved intestinal function and attenuated intestinal autophagy in β-CG-challenged piglets.	[192]
	Radiation-treated HaCaT cells	↓Beclin-1 ↓p62 ↓LC3 ↓Atg5	NAC treatment significantly inhibited radiation-induced autophagy in keratinocytes.	[193]
	Primary microglia cells isolated from cART-treated rats with HIV	↑Lamp2 ↑CTSD ↓LC3-II ↓SQSTM1	NAC reversed the damaging effects of cART.	[194]
	STZ-induced rats with T2DM subjected to myocardial I/RI	↓AMPKα ↓LC3-II/I ↓p62 ↓mTOR	NAC exerted cardioprotective effects primarily through inhibition of excessive autophagy.	[195]

Abbreviations: PQ—paraquat; AAC—abdominal aortic constriction; mHypoA-59—transformed murine hypothalamus neurons; β-CG—β-conglycinin; HaCaT—immortalized human keratynocytes cell line; cART—combined antiretroviral therapy; I/RI—ischemia/reperfusion injury.

**Table 9:** Summary of selected available data on the modulation of PUFAs-mediated autophagy.

Compound Used	Experimental Model	Signaling Mediators	Results	Ref.
ω-3 PUFA	Rats with TBI	↑LC3 ↑Beclin-1 ↑Atg3 ↑Atg7 ↑p62	ω-3 PUFA supplementation attenuated TBI-induced apoptosis by inducing autophagy through upregulation of the SIRT1-mediated deacetylation of Beclin-1.	[198]
ω-6 PUFA (linoleic acid)	*Larimichthys crocea* (in vivo), *Larimichthys crocea* hepatocytes in vitro	↑Beclin-1 ↑ULK1 ↑Atg101 ↑Atg12 ↑Atg4b ↑LC3 ↑p62	Linoleic acid induced autophagy through the AMPK/mTOR signaling pathway.	[199]
DHA, EPA	L02 cell line	↑LC3-II/I	DHA and EPA protected hepatocytes during lipotoxicity through the induction of autophagy.	[200]
	STZ-treated Fat-1 transgenic mice	↑LC3 ↓p62	Fat-1 modification protected against STZ-induced β cell death by the activation of autophagy.	[201]
	Fat-1 transgenic mice	↑LC3-II/I ↑Atg7 ↓p62	Fat-1 modification caused a reduction in the body weight and activation of autophagy in the hypothalamus.	[202]
	Purkinje cells of fat-1 transgenic mice with STZ-induced diabetes	↑LC3-II/I ↑Beclin-1 ↑p-Akt ↓p62	STZ-treated fat-1 mice were protected from Purkinje cell loss and exhibited increased BDNF signaling, which enhanced autophagy.	[203]
	Fat-1 transgenic mice with ConA-induced T cell-mediated hepatitis	↑LC3-II/I ↓p62	n-3 PUFAs limited ConA-induced hepatitis via an autophagy-dependent mechanism.	[204]
	Fat-1 transgenic mice with I/R-mediated renal injury	↑Beclin-1 ↑Atg7 ↑LC3-II/I ↑p-AMPK/AMPK ↓p62 (baseline lower than wt) ↓p-mTOR/mTOR	ω3-PUFAs in fat-1 mice contributed to AMPK-mediated autophagy activation, leading to a renoprotective response.	[205]
DHA	Bone marrow-derived macrophages from fat-1 transgenic mice	↑LC3-II/I ↑p-AMPKα	ω3-PUFAs and DHA-mediated control of *T. gondii* infection suggested that ω3-PUFAs might serve as a therapeutic candidate to prevent toxoplasmosis.	[206]
	D54MG, U87MG, U251MG, GL261 cell lines	↑LC3-II/I ↓p-Akt/Akt ↓p-mTOR/mTOR ↓p-AMPK/AMPK	DHA induced cell death through apoptosis and autophagy in glioblastoma cells.	[207]

Abbreviations: ConA—Concavalin A; wt—wild type; TBI—traumatic brain injury; SIRT1—Sirtuin 1; L02—Human papillomavirus-related endocervical adenocarcinoma cell line; DHA—docosahexaenoic acid; EPA—eicosapentaenoic acid; BDNF—brain-derived neurotrophic factor; ConA—concavalin A; D54MG, U87MG, U251MG, GL261—human glioblastoma cell lines of different origin.

## Data Availability

Not applicable.
